# Radiographic and Functional Results following Subtalar Arthroereisis in Pediatric Flexible Flatfoot

**DOI:** 10.1155/2019/5061934

**Published:** 2019-08-01

**Authors:** David Ruiz-Picazo, Plácido Jiménez-Ortega, Francisco Doñate-Pérez, Natalia Gaspar-Aparicio, Victor García-Martín, José Ramírez-Villaescusa, Sergio Losa-Palacios

**Affiliations:** Department of Pediatric Orthopaedics Surgery, Complejo Hospitalario Universitario de Albacete, Spain

## Abstract

**Introduction:**

Flexible flatfoot (FFF) is one of the most common skeletal disorders in children. In symptomatic patients who do not respond to conservative measures, surgery may be an option. Subtalar arthroereisis consists of limiting excessive eversion of the subtalar joint through different types of implants.

**Materials and Methods:**

We carried out a retrospective study of 16 patients (32 feet) intervened for FFF with a subtalar device (arthroereisis), across the period of 2008-2015 with a minimum follow-up period of one year. Pre- and postoperative measures of the Moreau-Costa-Bartani angle, dorsoplantar (DP) and lateral (L) talocalcaneal angle, talonavicular coverage angle, and naviculocuboid overlap were used to evaluate correction of the deformity. Two expert surgeons from the Pediatric Orthopedics Unit took separate measurements of these angles for subsequent analysis purposes and to obtain the interobserver correlation coefficient for quantitative variables. Pre- and postoperative differences in the measurement of angles were ascertained using Student's* t*-test for paired samples; and a functional evaluation of the patients intervened was carried out pre- and postoperatively by administering the parent version of the Oxford Ankle Foot Questionnaire for Children (OxAFQ-C) during a clinical interview. All statistical analyses were performed using the SPSS v. 19.0 program (SPSS, Chicago, IL), with values being deemed statistically significant at* p*<0.05.

**Results:**

A breakdown of the study population showed 81.25% (13 patients) males and 18.75% (3 patients) females, with mean age of 9 years (7-11). The interobserver coefficient for quantitative variables displayed a very high degree of concordance (0.7-0.9), indicating that the validity of the measures was adequate. Pre- and postoperative analysis of differences in the measurement of angles was statistically significant (p<0.005). In terms of functional evaluation, the postoperative results were positive, with statistical significance for the “school and play”, “emotional”, and “footwear” domains of the OxAFQ-C scale and no differences in the “physical” domain.

**Conclusion:**

Subtalar arthroereisis is a valid option for the treatment of symptomatic pediatric flatfoot, with good postoperative functional and radiographic results.

## 1. Introduction

Although flexible flatfoot (FFF) is one of the most common skeletal disorders among children, controversy surrounds the definition of pathological flexible flatfoot [1]. A habitual criterion of this condition is a very reduced or absent arch, excessive heal eversion during weight bearing, and forefoot abduction that causes a collapse of the foot. In children with FFF, the longitudinal arch is reconstructed when the child is standing on tiptoe or there is hyperextension of the hallux due to the fascia plantar windlass mechanism [2–4].

FFF seldom causes pain or disability in childhood [1]. Evaluation of these children at the physician's office is often due to parents' concern about the foot's appearance and/or excessive asymmetric shoe wear [5]. In a study of 242 children with FFF across a 3-year follow-up period, Coll Bosch et al. observed that pediatric FFF resolves spontaneously with growth, that the optimal age for diagnosis is from 5 to 6 years, and that treatment does not influence the natural evolution of flatfoot in the child [6].

Staheli et al. [7] describe a “typical” case of FFF as any asymptomatic pediatric patient who needs no specific treatment other than “wait and see” [1, 3]. If the clinical examination reveals an apparent shortening of the Achilles tendon, stretching exercises are indicated [1, 3]. Nonetheless, there have been reports that FFF, which leads to a retraction of the Achilles tendon, will inevitably worsen in adult life and become symptomatic [3]. MacKenzie et al. found very limited evidence of the effectiveness of conservative treatments in symptomatic patients [8].

Hence, in patients presenting with symptomatic FFF, which proves nonresponsive to conservative measures, surgical treatment may be considered.

The arthroereisis procedure has been described as a valid treatment option, which enables the internal plantar arch to be restored with few complications. Several procedures have been described, including subtalar extra-articular screw arthroereisis, the calcaneo-stop technique, and the subtalar implant [9–11]. This paper focuses on the last-mentioned procedure, which consists of placing a cylindrical device in the subtalar joint, with the aim of limiting eversion of the foot. Accordingly, this study sought to analyze any improvement, both radiologic and functional, seen in patients treated by means of this procedure.

## 2. Materials and Methods

We conducted a retrospective study of patients treated for symptomatic FFF with the arthroereisis technique, across the period of 2008-2015. Treated patients were intervened using a cannulated titanium subtalar device.

The inclusion criteria were defined as any child who (1) had idiopathic symptomatic FFF; (2) was aged 7 to 12 years; (3) had been intervened using subtalar arthroereisis without an associated surgical procedure (Achilles tendon lengthening or accessory navicular removal); (4) presented with no neuromuscular, neurogenic, or osseous anomalies; (5) had an appropriate pre- and postoperative radiologic study; and (6) had undergone a minimum of one-year follow-up. Patients with no valid radiologic study and/or a follow-up of less than one year were excluded.

All patients were intervened by general anaesthesia using lateral approach to subtalar joint. A curved 2 cm skin incision was made on the lateral side of the hindfoot over the sinus tarsi. A K-wire was placed on the subtalar joint and was checked with fluoroscopy. Then the trial implants (with increasing diameters) were inserted until the appropriate implant size was determined. The authors chose the smallest implant that corrected the deformity and remained stable in the sinus tarsi while moving the subtalar joint. Lastly, the definitive cannulated titanium device was placed in the subtalar joint.

Based on the parent version of the Oxford Ankle Foot Questionnaire for Children (OxAFQ-C) administered during a clinical interview, the clinical variables of age, sex, and functional status were evaluated preoperatively and again at the end of follow-up.

Radiologic study was performed in upright position. Dorsoplantar radiography is performed with the X-ray tube tilted 30 degrees from the vertical axis, in a distal-proximal (toe-to-heel) direction, at a distance of 90 cm from the foot. Lateral radiography was obtained by placing the X-ray tube parallel to the horizontal axis, at a distance of 90 cm from the foot. These angular and linear measurements were made with the aid of our institution's Radiologic Archive and Image Management (Raim-PC) computer software program.

**Figure 1:**
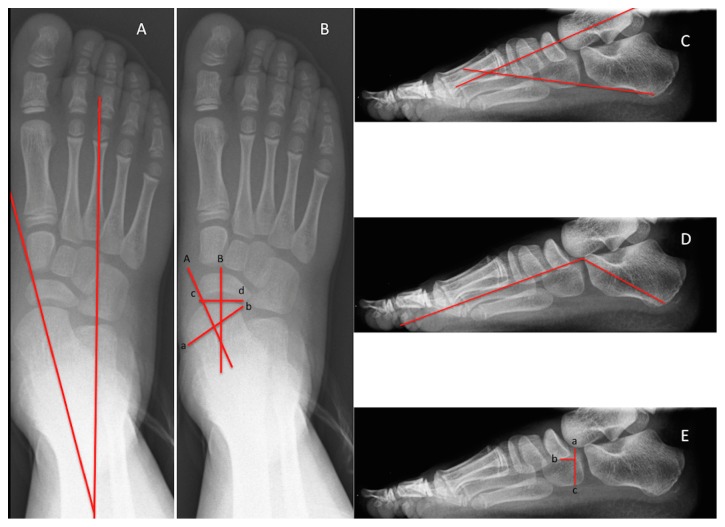
The graphical representation of angles measures used in the study. A: DP talocalcaneal angle. B: talonavicular coverage angle. Points a and b = medial and lateral edges (resp.) of the articular surface of the talus bone. Points c and d = medial and lateral edges (resp.) of the articular surface of the navicular bone. Line A is perpendicular to ab, and line B is perpendicular to cd. Talonavicular coverage is the angle created by the intersection of lines A and B. C: talocalcaneal angle in lateral radiography. D: Moreau-Costa-Bartani angle. E: naviculocuboid overlap. Points a and b = superior and inferior margins (resp.) of the cuboids. Point c = inferior margin of the navicular. Naviculocuboid overlap = ac/ab X 100.

To evaluate the degree of correction, the following angles were measured in pre- and postoperative study: (1) talonavicular coverage angle, (2) naviculocuboid overlap, (3) talocalcaneal angle in lateral radiography, (4) talocalcaneal angle in dorsoplantar (DP) radiography, and (5) the Moreau-Costa-Bartani angle (Figure 1).

The* talonavicular coverage angle* is the angle formed by the perpendicular to the line joining the medial and lateral edges of the articular surface of the talus bone and the perpendicular to the line joining the medial and lateral edges of the articular surface of the navicular bone. Davids et al. [12] established normal values for this angle, that is, 20° (±9.8) with a range of 5°-39°. High degrees of coverage are related to greater midfoot abduction.* Naviculocuboid overlap* is measured by dividing the product of the superior margin of the cuboid bones and inferior margin of the navicular bone by the product of the superior and inferior margins of the cuboids, expressed as a percentage. Naviculocuboid overlap indicates greater eversion, that is, collapse of the foot. In the study by Davids et al. [12], the normal values were 47% (±13.8) with a range of 22%-85%. In our study, the results were 66.43% (range: 52.37%-80.49%) in the right foot and 57.8% (range: 47.51%-68.11%) in the left foot. The* lateral talocalcaneal* angle is obtained from the intersection between the line drawn from the mid-talar axis and that joining the plantar prominence of the calcaneus to the calcaneocuboid surface, measured on lateral projection radiography. The normal values for this angle are 39° to 49° (range: 36°-61°) [13]. This angle evaluates hindfoot valgus and abduction (greater the angle, the greater the degree of hindfoot abduction and valgus). The* talocalcaneal angle* (Kite's angle) refers to the angle between lines drawn down the axis of the talus and calcaneus measured on a weight-bearing DP foot radiography. The talocalcaneal angle should measure between 25 and 40 degrees. Lastly,* the Moreau-Costa-Bartani* is the angle formed by the following lines: line formed from the lower point of the medial sesamoid to the lower point of the talonavicular joint and the line formed from the lower point of the talonavicular joint to the lower point of the posterior calcaneal tuberosity. The normal values for this angle are 115°-125°.

The statistical analysis consisted of describing the variables and analyzing the clinical (OxAFQ-C) and radiologic differences, both preoperatively and at the end of follow-up, using Student's* t*-test for paired samples. To establish the degree of reliability of their measurements, two expert surgeons from the Pediatric Orthopedics Unit took separate measurements of the angles and then used the intraclass correlation coefficient of quantitative variables to establish the degree of interobserver correlation according to the Fleiss scale [14].

All statistical analyses were performed using the SPSS v. 19.0 (SPSS, Chicago, IL) computer software program, with values being deemed statistically significant at* p*<0.05.

## 3. Results

Of the initial 22 patients (44 feet), 16 (32 feet) were included in the study, with 6 patients being excluded due to the absence of a valid radiologic study. A breakdown of the study population showed 81.25% males (13 patients) and 18.75% females (3 patients), with mean age of 9 years (range: 7 to 11 years).

The preoperative clinical study evaluated with Student's* t*-test indicated significant differences in OxAFQ-C scale scores in the “school and play”, “emotional”, and “footwear” domains (*p*<0.05) but no differences in the “physical” domain (Table 1).

The radiologic study showed significant differences, evaluated preoperatively and again during follow-up (minimum one year), in the degree of correction of all angles in the dorsoplantar and lateral planes (*p*<0.05) (Table 2).

The degree of interobserver correlation for radiologic measurements was substantial or almost perfect, both preoperatively and at the end of follow-up (Table 3). In terms of functional evaluation (Table 3), the postoperative results were positive, displaying statistical significance for the “school and play”, “emotional”, and “footwear” domains of the OxAFQ-C (parent version) scale.

The implants were not removed because the patients had not yet achieved bone maturity.

Lastly, there were 4 cases of complications related with overcorrection of the foot and expulsion of the implant due to erroneous measurement.

## 4. Discussion

FFF is a common disorder characterized by plantar flexion and medial rotation of the talus, calcaneal eversion, medial longitudinal arch collapse, and abduction of the forefoot [5, 6, 15]. Its incidence is unknown but is estimated to be close on 5% among children and adults [16].

Pediatric FFF is often asymptomatic, and conservative treatment begins by using new footwear and ortheses. In cases where FFF is symptomatic and conservative modalities have failed, surgical intervention is indicated and in some patients arthroereisis may be considered.

Subtalar arthroereisis is a surgical procedure aimed at placing an implant in the sinus tarsi to limit excessive eversion of the subtalar joint. In 1946, Chambers et al. discussed this technique by describing the placement of a bone graft in the posterior subtalar joint to restrict hindfoot eversion [17]. Even so, subtalar arthroereisis continues to be controversial, especially in terms of indications, age at date of surgery, and adjunct procedures [18, 19].

Subtalar implants have been classified by reference to their biomechanical properties, for example, self-locking wedge, axis-altering, and impact-blocking devices [20]. Currently, most subtalar implants are of the self-locking wedge type, aimed at restricting hindfoot valgus. All our patients were intervened by placement of a titanium self-locking wedge device.

**Figure 2:**
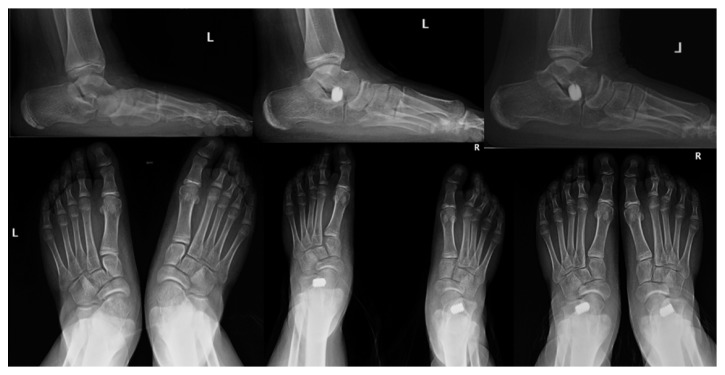
Male patient aged 11 years with bilateral flatfoot, showing foot remodeling trends after subtalar implant. The images in the centre were taken two months after operation; the images on the right correspond to two years after operation.

Patient age is a very important factor when it comes to the precise timing of the intervention [21]. It is recommended that surgery be performed between the ages of 8 and 12 years: all our patients were intervened at ages of 7 to 11 years. This is justified because, before the age of 8, many children may experience spontaneous correction. On the other hand, the performance of surgery beyond the age of 12 years tends to be exceptional, given that the aim of arthroereisis is to reposition the talus correctly on the calcaneus to enable remodeling of these bones and the subtalar joint during growth. Since at least 2 years are thought to be required for the purpose, this means that, after 12 years of age, there is insufficient time for the bones and ligaments of the hindfoot to be remodeled [22] (Figure 2).

Most studies that evaluate the functional outcomes of intervened patients classify such patients according to whether or not they present with postoperative symptoms. On observing the variability of symptoms among patients, Benedetti et al. stated that binary classification was inadequate [23]. An alternative approach is to carry out a continuous evaluation of the effect that symptoms have on these children's quality of life. The OxAFQ-C is a validated questionnaire that was drawn up to evaluate quality of life in this population [24]. This instrument not only evaluates disease burden but can also be used to record clinical change over time. In our study, parents perceived a significant improvement in the “school and play”, “emotional”, and “footwear” domains. The “physical” domain scores did not prove to be statistically significant. Morris et al. found that children with FFF had a worse quality of life than did children with normal feet [24]. The principal differences between groups were found to be in the dorsoplantar plane, where children with FFF presented with greater midfoot eversion and supination of the forefoot during gait. Both factors correlated with worsening of their quality of life. In addition, a recent OxAFQ-C-based study published by Martinelli et al., with more than 10 years of follow-up, evaluated other tests designed to ascertain the effect of subtalar implants on children's physical activity [25]. These authors observed that while surgery did not alter the duration, frequency, or type of sports activities engaged in by children, it did alter their participation in such activities, emotional state, and footwear problems. Given the time of follow-up, the authors attributed the above findings to changes in growth stage (child to adolescent) and priorities of life. In addition, Faldini et al. used self-reported questionnaires to evaluate patient-perceived quality of life after subtalar arthroereisis with a bioabsorbable implant. Patients reported a high degree of satisfaction, without perceiving their quality of life as being compromised [26]. Our findings are consistent with most of the literature on subtalar arthroereisis outcomes [27, 28].

The Moreau-Costa-Bartani angles and talocalcaneal or Kite angle (Figure 1) has been used in the past to evaluate the presence of flatfoot in lateral and dorsoplantar projections. In our study, these angles improved significantly after intervention to the point where they came to lie within their physiologic range. More recently, the talonavicular coverage angles [12] (Figure 1), previously described by Sangeorzan et al. [29], naviculocuboid overlap [12] (Figure 1), expressed in percentage terms, and the talocalcaneal angle in lateral radiography have been proposed for obtaining an overall evaluation of pediatric flatfoot. A significant postoperative improvement in talonavicular coverage angle (*p*<0.001) has been related to a reduction in midfoot abduction. Naviculocuboid overlap has been linked by Davids et al. to midfoot eversion [12]. In this latter study, normal values were 47 ± 13.8. In our study, the preoperative values of this angle were high and were related to greater midfoot eversion. With respect to the lateral talocalcaneal angle, the greater the angle, the greater the degree of hindfoot valgus and abduction. The literature shows that normal values range from 49 (±6.9) (36–61) [10] to 39 (±7) [1]. The values registered in our study are similar to those reported in the literature (left foot: 44.68 (41.12 – 48.25); right foot: 40.5 (37.04 – 43.95)), with the postoperative results proving significant.

**Figure 3:**
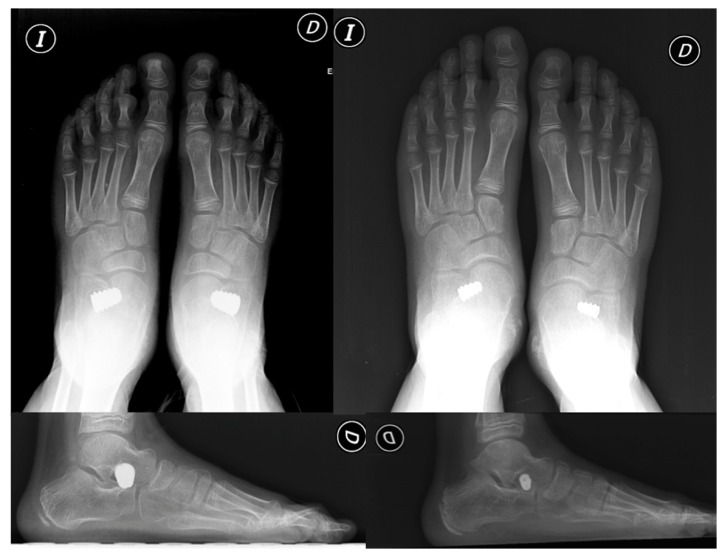
Example of overcorrection and expulsion of the implant. Revision surgery and placement of smaller-sized implants were necessary.

Insofar as complications are concerned, mention might be made of the more general types of complications, such as persistent pain in the sinus tarsi, overcorrection or undercorrection, and incorrect choice of size of implant, which may lead to its extrusion [30]. In our study, there were two cases (6.25%) in which extrusion of the implant due to incorrect choice of size was detected (Figure 3). In their study, Cook et al. established that a reduced postoperative talocalcaneal angle on AP X-ray protected against extrusion of the implant [31]. In addition, overcorrection was observed in two of the patients in the sample (6.25%). In these cases, the implant was changed with good results. There have been reports of implant-related complications closely linked to the materials used in their design, for example, wear, reaction to foreign bodies, and fracture [12, 13, 20, 21, 29, 30]. Furthermore, the following have also been documented in the literature: presence of intraosseous cysts in the talus bone, osteonecrosis of the talus, contraction of the peroneal muscles, and fractures of the calcaneus or talus [32–34].

The radiographic data on and subjective results of subtalar arthroereisis in FFF have been promising for children and adults alike. A review of data in the last decade showed a satisfaction rate of 81% to 90% among pediatric patients [17, 19, 22, 27].

## 5. Conclusion

Subtalar arthroereisis is a valid option for treatment of symptomatic pediatric flatfoot, with good postoperative radiographic results. We introduced measures for some angles which, though not commonly used, are nonetheless helpful when it comes to obtaining a more accurate preoperative evaluation of candidate patients. The talonavicular coverage angle furnished information on midfoot abduction; naviculocuboid overlap made it possible to assess eversion of the foot; that is, the greater the overlap, the greater the collapse of the foot; and the talocalcaneal angle in lateral radiography furnishes information on hindfoot valgus. Lastly, functional analysis of patients shows that surgery improves their participation in school and physical activities, emotional state, and footwear problems.

## Figures and Tables

**Table 1:** Pre- and postoperative differences in the measurement of angles. All values are statistically significant.

	Preoperative				Postoperative				Difference in means	
	Left foot	95% CI	Right foot	95% CI	Left foot	95% CI	Right foot	95% CI	Left foot (p value)	Right foot (p value)
Costa-Bartani angle	144.43	138.84 - 150.03	143.31	139.29 - 147.34	133.62	129.13 - 138.11	131.5	127.61 - 135.39	<0.001	<0.001

AP talocalcaneal angle	28.69	25.05 - 32.32	29.06	26.6 - 31.52	17.43	13.89 - 20.98	16.06	13.54 - 18.58	<0.001	<0.001

Lateral talocalcaneal angle	44.68	41.12 - 48.25	40.5	37.04 - 43.95	35	32.18 - 37.81	34.56	31.53 - 37.59	<0.001	0.002

Talonavicular coverage angle	31.18	25.77 - 36.6	34.12	29.71 - 38.53	14.37	8.78 - 19.96	16.12	11.18 - 21.06	<0.001	<0.001

Naviculocuboid overlap (%)	66.43	52.37 - 80.49	57.81	47.51 - 68.11	40.31	28.8 - 51.82	32.37	23.06 - 41.69	0.003	<0.001

**Table 2:** Degree of interobserver correlation. It will be noted that measurements range from 0.8 to close on 1, indicating a very high degree of correlation.

	Preoperative		Postoperative	
	Right foot	Left foot	Right foot	Left foot
Costa-Bartani angle	0.97	0.975	0.82	0.93

AP talocalcaneal angle	0.745	0.859	0.928	0.836

Lateral talocalcaneal angle	0.876	0.860	0.849	0.869

Talonavicular coverage angle	0.759	0.9	0.97	0.991

Naviculocuboid overlap	0.934	0.994	0.973	0.968

**Table 3:** Mean pre- and postoperative differences in the parent version of the Oxford Ankle Foot Questionnaire for Children (OxAFQ-C).

Parent version of the OxAFQ-C score (mean )			
OxAFQ-C domains	Preoperative	Postoperative	*P* value

Physical	63.5	71.2	0.2

School and play	87.6	91.7	<0.05

Emotional	90.5	92.6	<0.05

Footwear	68.3	81.1	<0.05

## Data Availability

The statistical analyses used to support the findings of this study are included within the article.
